# Filanesib plus bortezomib and dexamethasone in relapsed/refractory t(11;14) and 1q21 gain multiple myeloma

**DOI:** 10.1002/cam4.4451

**Published:** 2021-12-17

**Authors:** Darren Pan, Jonathan L. Kaufman, Myo Htut, Manish Agrawal, Amitabha Mazumder, Robert F. Cornell, Jeffrey A. Zonder, Joseph W. Fay, Manuel R. Modiano, Erin L. Moshier, Selena A. Rush, Brian J. Tunquist, Ajai Chari

**Affiliations:** ^1^ Tisch Cancer Institute Icahn School of Medicine at Mount Sinai New York New York USA; ^2^ Winship Cancer Institute Emory University Atlanta Georgia USA; ^3^ City of Hope National Medical Center Duarte California USA; ^4^ Maryland Oncology Hematology Columbia Maryland USA; ^5^ The Oncology Institute of Hope and Innovation Glendale California USA; ^6^ Vanderbilt University Medical Center Nashville Tennessee USA; ^7^ Karmanos Cancer Institute Detroit Michigan USA; ^8^ Texas Oncology Baylor Charles A Sammons Cancer Center Dallas Texas USA; ^9^ Arizona Clinical Research Center Hematology Oncology Tucson Arizona USA; ^10^ Department of Population Health Science and Policy Icahn School of Medicine at Mount Sinai New York New York USA; ^11^ Pfizer New York New York USA

**Keywords:** chemotherapy, clinical cancer research, clinical trials, experimental, medical oncology, multiple myeloma, therapeutics

## Abstract

Filanesib is a first‐in‐class kinesin spindle protein inhibitor which demonstrated safety and encouraging activity in combination with bortezomib and dexamethasone in relapsed/refractory multiple myeloma in a preliminary analysis of dose‐escalation phase results. This multicenter study included first a dose‐escalation phase to determine maximum tolerated dose of two schedules of filanesib, bortezomib, and dexamethasone and a subsequent dose‐expansion phase using the maximum tolerated doses. In the dose‐expansion phase, 28 patients were evaluable for safety and efficacy. The most common grade ≥3 adverse events were neutropenia (21%) and anemia (18%), which were noncumulative and reversible, and hypertension (18%). The overall response rate was 43% with median duration of response not yet reached (range, 2.8–23.7+ months) with median follow‐up of 6.3 months. A post hoc analysis incorporated 29 dose‐escalation phase patients who received therapeutic filanesib doses, with an overall response rate of 39% and median duration of response of 18.0 months among the 57 total patients with median progression‐free survival of 8.5 months. Notably, the PFS of high risk patients was comparable at 8.5 months, driven by the patients with 1q21 gain, characterized by increased MCL‐1 expression, with a PFS of 9.1 months versus 3.5 months for the remainder of high risk patients. Patients with t(11;14) also had an encouraging PFS of 15.0 months. The combination of filanesib, bortezomib, and dexamethasone continues to show safety and encouraging activity in relapsed/refractory multiple myeloma, particularly in those patients with 1q21 gain and t(11;14).

## Graphical abstract

We report on the final analysis of the phase 1 trial of filanesib, bortezomib, and dexamethasone in relapsed/refractory multiple myeloma. Among patients receiving therapeutic doses of the study drugs, overall response rate was 39% with median duration of response of 14.1 months and median progression‐free survival of 8.0 months. The combination continues to show safety and encouraging activity in this population, particularly in those patients with 1q21 gain and t(11;14).

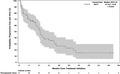

## 1 INTRODUCTION

Multiple myeloma treatment has been improved materially with the introduction of immunomodulating agents (IMiDs), proteasome inhibitors (PIs), and monoclonal antibody therapy, yet the disease remains largely incurable. Now many efforts have turned toward agents with novel mechanisms such as kinesin spindle protein (KSP) inhibitors. KSP is a mitotic spindle motor protein essential for mitosis.^1^, ^2^ KSP inhibitors produce prolonged mitotic arrest during which protein synthesis is halted, leading to depletion of proteins such as the apoptosis inhibitor myeloid cell leukemia 1 (MCL‐1).^3^ Neoplastic plasma cells depend on MCL‐1 as a survival signal and undergo apoptosis when treated with KSP inhibitors.^2^, ^4^

Filanesib (ARRY‐520) is a highly selective, first‐in‐class KSP inhibitor. A phase 1 study demonstrated tolerability of single‐agent filanesib in relapsed or refractory multiple myeloma (RRMM) at a maximum tolerated dose (MTD) of 1.50 mg/m^2^/day.^5^ Using this MTD in phase 2, the investigators confirmed the presence of modest single‐agent activity with an overall response rate (ORR) of 16% and clinical benefit rate (CBR, responses of ≥minimal response, MR) of 23% in patients with a median of over six previous lines of therapy. Although the median progression‐free survival (PFS) was only 1.6 months, responses were durable, with a median of 8.6 months. With evidence of single‐agent activity, filanesib was next investigated as part of combination therapy.

In preclinical models, filanesib combined with bortezomib showed synergistic apoptotic activity and remained highly active in cell lines resistant to bortezomib.^6^ The efficacy of the filanesib–bortezomib combination may be mediated in part by increased levels of a MCL‐1 fragment which promotes apoptosis.^6^ Furthermore, inactivation of MCL‐1 by filanesib can sensitize multiple myeloma cells to dexamethasone.^2^, ^7^ These preclinical data paved the way for the rational combination of filanesib with dexamethasone and bortezomib in clinical settings.

The safety of the combination of filanesib, bortezomib, and dexamethasone was investigated in a phase 1 dose‐escalation study in RRMM.^8^ Exacerbated neutropenia was observed in the first two dose‐escalation cohorts, possibly related to the crucial role of MCL‐1 in neutrophil development and survival.^9^, ^10^ Subsequently, prophylactic subcutaneous injections of granulocyte‐stimulating factor (G‐CSF) were included in the protocol for 5–7 days after each filanesib dose. Filanesib doses of 1.50 mg/m^2^ in schedule 1 (administered on days 1, 2, 15, and 16) and 3 mg/m^2^ in schedule 2 (administered on days 1 and 15) were determined to be the MTDs. Grade 3/4 neutropenia, thrombocytopenia, and anemia occurred in 44%, 29%, and 29% of patients, respectively, whereas the most common nonhematologic grade 3/4 toxicities were lipase elevation (11%), amylase elevation (7%), and pneumonia (7%). Hematologic toxicities were noncumulative and reversible upon the addition of prophylactic G‐CSF. Despite patients having received a median of three lines of prior therapy and 56% of patients with PI‐refractory disease, an ORR of 20% was attained with a median duration of response (DOR) of 14.1 months. Within the subset of patients given therapeutic doses of filanesib (≥1.25 mg/m^2^) and bortezomib (1.3 mg/m^2^), an ORR of 40% was attained with DOR of 17.2 months. An ORR of 29% was seen among PI‐refractory patients receiving therapeutic dosing.

Prior data have also investigated the use of filanesib as a personalized treatment option for multiple myeloma. Both the single agent and combination trials suggested that alpha 1‐acid glycoprotein (AAG) may serve as a biomarker for predicting filanesib response, with *in vitro* data showing that AAG binds filanesib and lowers circulating levels of free drug.^11^ With filanesib monotherapy, all responders had AAG <110 mg/dl, while the combination therapy trial noted a trend toward longer time on study (ToS) with lower AAG.^5^, ^8^ Filanesib's activity in 1q21 gain patients, which had not yet been elucidated, is another area of interest given the higher MCL‐1 expression in this high‐risk subset.^12^, ^13^ To date, the efficacy venetoclax in t(11;14) carriers stands as a rare example of myeloma therapy targeted to such a cytogenetic subtype.^14^ New therapies for 1q21 gain patients are particularly vital given their poorer responses to both standard triplet regimens and daratumumab.^15^, ^16^

Based on the safety and encouraging preliminary efficacy of filanesib plus bortezomib and dexamethasone, two dose‐expansion schedules of this triplet regimen have been conducted in RRMM; one schedule administered filanesib on cycle days 1, 2, 15, and 16 while a second more convenient schedule administered higher doses of filanesib on days 1 and 15. Here, we report on the efficacy, safety, and pharmacokinetic results of the dose‐expansion phase.

## 2 MATERIALS AND METHODS

### 2.1 Study design

This was a phase 1 multicenter study with a dose‐escalation phase and a subsequent dose‐expansion phase. The dose‐escalation phase determined the MTD of two schedules of filanesib and bortezomib with and without dexamethasone. The dose‐expansion phase was planned to obtain preliminary estimates of the efficacy. The design was motivated by the operating characteristics when using a Simon two‐stage design with a null hypothesis of >50% ORR (85% power, *α* = 0.15). With such a design, 21 evaluable patients would be needed for the first stage with >7 responders needed to move to stage 2. The development plan contemplated conducting the stage 2 portion (if applicable) as a separate study. Additional objectives were to further evaluate the drug combination's safety to assess pharmacokinetic interactions between filanesib and bortezomib, and to explore possible biomarkers of response including AAG. Data analyses focused on the previously unreported dose‐expansion phase with secondary analyses of the composite of patients from both phases treated with therapeutic doses of study drugs.

### 2.2 Patients

Patients were eligible for participation if ≥18 years of age with measurable RRMM or plasma cell leukemia. Patients in the dose‐escalation phase had received two or more lines of prior treatment including an IMiD and a PI, with progression of disease (PD) during the last prior regimen or after. Patients in the dose‐expansion phase had received between one and three lines of prior treatment, but patients with bortezomib‐refractory disease were excluded. Patients had an Eastern Cooperative Oncology Group performance status of either 0 or 1, adequate liver function, serum creatinine ≤2.5 mg/dl or calculated creatinine clearance ≥50 ml/min, a neutrophil count ≥1.5 × 10^9^/L and a platelet count ≥75 × 109/L (or ≥50 × 10^9^/L if bone marrow contained ≥50% plasma cells) without transfusion or growth factor support for 2 weeks before screening. For both phases, exclusion criteria included a diagnosis of primary amyloidosis or stem cell transplantation performed within 3 months before initiating study treatment.

The study followed the International Conference on Harmonisation of Technical Requirements for Registration of Pharmaceuticals for Human Use Good Clinical Practice, obtained approval from the Institutional Review Boards of each participating center, and obtained written informed consent from patients. This study was registered at www. ClinicalTrials.gov (NCT01248923).

### 2.3 Treatment schedules

In schedule 1, 1.50 mg/m^2^/day of filanesib was administered on days 1, 2, 15, and 16 of 28‐day cycles with 1.3 mg/m^2^/day dose of bortezomib subcutaneously and 40 mg/day of dexamethasone orally on days 1, 8, and 15 with prophylactic G‐CSF for 5–7 days on day 3 or 4 and day 17 or 18. In schedule 2, 3.0 mg/m^2^/day of filanesib was administered on days 1 and 15 of each cycle with 1.3 mg/m^2^ of bortezomib plus 40 mg/day of dexamethasone orally on days 1, 8, and 15 with prophylactic G‐CSF for 5–7 days on day 2 or 3 and day 16 or 17. Bortezomib could be administered intravenously if investigators believed it was in the patient's best interest. In both schedules, patients ≥75 years were given 20 mg/day of dexamethasone. Patients continued to receive study therapy in repeating cycles until unacceptable toxicity or PD as long as the investigator deemed appropriate for the patient's care. Varicella zoster virus prophylaxis was prescribed per standard of care, and antibiotic prophylaxis against gram‐negative organisms was prescribed for neutropenic patients.

### 2.4 Drug concentration measurements

For expansion‐phase patients, venous blood samples were drawn before each filanesib infusion and 5 h after initiating the infusion during the first cycle. These blood samples were sent for plasma determination of filanesib and bortezomib levels and AAG concentrations.

### 2.5 Assessments

Safety was assessed based on adverse events (AEs), laboratory tests, and electrocardiograms. AE severity was assessed using the National Cancer Institute Common Terminology Criteria for Adverse Events 4.0 and coded with the Medical Dictionary for Regulatory Activities 13.0.

Investigators used the International Myeloma Working Group response criteria in determining ORR.^17^ DOR was defined as time from partial response (PR) or better to the date of first progression or death. ToS was defined as the time from first dose of the study drugs to date of study termination. Post hoc analyses included minimal response (MR), clinical benefit rate (CBR), and disease control rate (DCR). CBR was calculated as the percentage of patients with response greater than or equal to MR; the DCR included patients with response greater or equal to stable disease for ≥8 weeks. High‐risk cytogenetics was considered to be ≥1 of del(17p), t(14;16), or 1q21 gain.

### 2.6 Statistical analysis

In the expansion phase, cohorts of up to approximately 21 evaluable patients were anticipated to be enrolled. Continuous variables were summarized by the median (range: minimum‐maximum) and categorical variables by *N* (%). Patients were evaluable for safety and efficacy if they received at least 1 dose of filanesib. The Kaplan–Meier method was utilized to estimate PFS and DOR with patients censored at the last date known to be alive and progression free. No formal comparisons were planned or performed.

To assess for drug interactions, comparisons of plasma drug concentrations of filanesib and bortezomib were assessed as geometric mean ratios by patient and day. Results for ratios were presented for each patient and summarized using descriptive statistics. The effects of concomitant bortezomib administration on filanesib concentrations and vice‐versa were assessed using analysis of variance. SAS 9.4 was used for statistical tests.

## 3 RESULTS

### 3.1 Patient population

In the dose‐expansion phase, 7 patients in schedule 1 and 21 in schedule 2 were enrolled and dosed between November 5, 2013 and December 3, 2014 at six centers in the United States. Enrollment began with schedule 2 due to ease of dosing. After 21 patients were accrued, schedule 1 enrollment began. Due to slowing enrollment at this time, enrollment was closed after seven patients. With a total 28 patients between the two schedules, it was determined that estimates of response rates would not be meaningfully affected. At the time of data cutoff (November 21, 2016), four patients who were responding to treatment at study closure were transitioned to a rollover study to continue receiving the combination.

Table 1 details demographic data. The median age of the patients was 63 in schedule 1 and 69 in schedule 2 (ranges 47–66 and 53–79, respectively) and patients had a median of 2 and 3 prior systemic lines of therapy, respectively (ranges 1–3 and 1–4, respectively). Across the two schedules together, 71% of patients had received prior stem cell transplantation, 86%t had received a PI, and 89% had received an IMiD.

**TABLE 1:** Demographic and baseline disease characteristics and prior therapies

	Dose‐escalation		Dose‐expansion		Phase 1 therapeutic total
Characteristic	Schedule 1, therapeutic (*N* = 19)	Schedule 2 (*N* = 10)	Schedule 1 (*N* = 7)	Schedule 2 (*N* = 21)	(*N* = 57)
Sex, no. (%)					
Male	8 (42)	3 (30)	0 (0)	11 (52)	29 (51)
Female	11 (58)	7 (70)	7 (100)	10 (48)	28 (49)
Race, no. (%)					
American Indian	0 (0)	0 (0)	1 (14)	0 (0)	1 (2)
Asian	1 (5)	0 (0)	1 (14)	2 (10)	4 (7)
White	15 (79)	7 (70)	4 (57)	10 (48)	36 (63)
Black/African American	3 (16)	3 (30)	1 (14)	9 (43)	16 (28)
Age at consent (years)					
Median (range)	64 (31–78)	65.5 (55–79)	63 (47–66)	69 (53–79)	65 (31–79)
Ig subtype at diagnosis, no. (%)					
IgG	3 (16)	3 (30)	5 (71)	12 (57)	35 (61)
IgA	12 (63)	6 (60)	1 (14)	5 (24)	12 (21)
IgM	1 (5)	0 (0)	0 (0)	0 (0)	1 (2)
Light chain only	3 (16)	1 (10)	1 (14)	4 (19)	9 (16)
Light chain at diagnosis					
Kappa	14 (74)	4 (40)	3 (43)	14 (67)	35 (61)
Lambda	5 (26)	6 (60)	4 (57)	7 (33)	22 (39)
ISS stage at diagnosis, no. (%)					
I	9 (47)	3 (30)	2 (29)	9 (43)	23 (40)
II	10 (53)	6 (60)	2 (29)	8 (38)	26 (45)
III	0 (0)	1 (10)	3 (43)	3 (14)	7 (12)
Missing	0 (0)	0 (0)	0 (0)	1 (5)	1 (2)
ECOG performance status, no. (%)					
0	8 (42)	2 (20)	2 (29)	6 (29)	18 (32)
1	11 (58)	8 (80)	4 (57)	15 (71)	38 (67)
2	0 (0)	0 (0)	1 (14)	0 (0)	1 (2)
Creatinine clearance, ml/min					
>90	6 (32)	4 (40)	4 (57)	3 (14)	17 (30)
61–90	8 (42)	3 (30)	2 (29)	7 (33)	20 (35)
31–60	4 (21)	3 (30)	1 (14)	10 (48)	18 (32)
<31	1 (5)	0 (0)	0 (0)	1 (5)	2 (4)
High‐risk cytogenetics, no. (%)^a^					
Yes	5 (26)	4 (40)	1 (14)	6 (29)	16 (28)
No	14 (74)	5 (50)	6 (86)	15 (71)	40 (70)
N/A	0 (0)	1 (10)	0 (0)	0 (0)	1 (2)
Prior lines of therapy, median (range)^b^	5 (3–10)	4 (2–11)	2 (1–3)	3 (1–4)	3 (1–11)
Prior proteasome inhibitor	19 (100)	10 (100)	6 (86)	17 (81)	52 (91)
Refractory	10 (53)	4 (40)	0 (0)	4 (19)	18 (32)
Prior bortezomib	17 (89)	9 (90)	6 (86)	15 (71)	47 (82)
Refractory	8 (42)	3 (30)	0 (0)	0 (0)	11 (19)
Prior carfilzomib	5 (26)	2 (20)	1 (14)	5 (24)	13 (23)
Refractory	5 (26)	2 (20)	0 (0)	4 (19)	11 (19)
Prior IMiD	19 (100)	10 (100)	6 (86)	19 (90)	54 (95)
Refractory	15 (79)	9 (90)	2 (29)	12 (57)	38 (67)
Prior thalidomide	3 (16)	0 (0)	1 (14)	7 (33)	11 (19)
Refractory	1 (5)	0 (0)	1 (14)	0 (0)	2 (4)
Prior lenalidomide	19 (100)	9 (90)	5 (71)	19 (90)	52 (91)
Refractory	14 (74)	8 (80)	1 (14)	11 (52)	34 (60)
Prior pomalidomide	3 (16)	4 (40)	1 (14)	4 (19)	12 (21)
Refractory	3 (16)	4 (40)	0 (0)	4 (19)	11 (19)
Refractory to PI and IMiD	7 (37)	4 (40)	0 (0)	3 (14)	14 (25)
Prior transplant	17 (89)	8 (80)	6 (86)	14 (67)	45 (79)

Abbreviations: ECOG, Eastern Cooperative Oncology Group; Ig, immunoglobulin; IMiD, immunomodulatory agent; ISS, International Staging System.

^a^ Defined as ≥1 of the following: del(17p), t(14;16), or 1q21 gain.

^b^ The dose‐escalation phase allowed bortezomib‐refractory patients, contributing to higher median lines of prior therapy.

Half of patients were IMiD‐refractory, 14% were PI‐refractory with carfilzomib, and 11% were refractory to both PI and IMiD. A quarter of patients had high‐risk cytogenetics.

### 3.2 Treatment exposure and safety

Patients received a median of four cycles of filanesib in schedule 1 and seven cycles in schedule 2 (ranges 1–15 and 1–26 cycles, respectively) with a median time on treatment of 3.7 months (range 0.9–14.3 months) and 6.4 months (range 0.9–25.8 months), respectively. The primary reasons for treatment discontinuation were PD (*n* = 14, [50%]), investigator decision (4, [14%]), toxicity (4, [14%]), and withdrawal of consent (2, [7%]). Four patients (14%) responding well to treatment were transitioned to a rollover protocol to allow continued treatment with study drug.

The safety profile of filanesib was comparable between schedules 1 and 2. All 28 patients experienced at least one AE, and 21 (75%) patients experienced an AE attributed to filanesib. The most common nonhematologic AEs were diarrhea (43%), peripheral neuropathy (39%), and pyrexia and fatigue (25% each). Of note, most patients had peripheral neuropathy at study initiation, and thus only 21% of patients experienced peripheral neuropathy assessed as related to filanesib or bortezomib.

Just over half the patients experienced a hematologic AE. All grade/grade ≥3 hematologic AEs (Table 2) included anemia (36%/18%), neutropenia (25%/18%), and thrombocytopenia (21%/14%). These events were rapidly reversible and did not appear to be cumulative. Seven percent of patients experienced febrile neutropenia. The most frequent infection was pneumonia, which occurred in two patients (7%). Bleeding events were rare and occurred in only one patient who experienced rectal bleeding, vaginal bleeding, and uterine bleeding in the setting of thrombocytopenia. Patients with creatinine clearance <60 ml/min (*n* = 12) had higher rates grade ≥3 hematologic AEs, most commonly neutropenia (35%), anemia (25%), and thrombocytopenia (25%), though we did not observe greater rates of clinical sequelae such as infections and bleeding.

**TABLE 2:** Treatment‐emergent grade ≥3 AEs occurring in >1 patient (safety population)

MedDRA‐preferred term, no. (%)	Dose‐escalation		Dose‐expansion		Phase 1 therapeutic total
	Schedule 1, therapeutic (*N* = 19)	Schedule 2 (*N* = 10)	Schedule 1 (*N* = 7)	Schedule 2 (*N* = 21)	(*N* = 57)
Nonhematologic AEs					
Lipase increase	4 (21)	2 (20)	1 (14)	0 (0)	7 (12)
Blood amylase increase	2 (11)	2 (20)	1 (14)	0 (0)	5 (9)
Hypertension	0 (0)	0 (0)	1 (14)	4 (19)	5 (9)
Pneumonia	0 (0)	1 (10)	1 (14)	1 (5)	3 (5)
Hypoxia	0 (0)	0 (0)	1 (14)	1 (5)	2 (4)
Hypokalemia	0 (0)	0 (0)	0 (0)	2 (10)	2 (4)
Acute renal failure	0 (0)	0 (0)	0 (0)	2 (10)	2 (4)
Fall	0 (0)	1 (10)	1 (14)	0 (0)	2 (4)
Hematologic AEs					
Neutropenia^a^	7 (37)	5 (50)	1 (14)	5 (24)	18 (32)
Anemia	3 (16)	4 (40)	1 (14)	4 (19)	12 (21)
Thrombocytopenia	3 (16)	3 (30)	0 (0)	4 (19)	10 (18)
Febrile Neutropenia^a^	1 (5)	0 (0)	0 (0)	2 (10)	3 (5)

Abbreviations: AE, adverse events; MedDRA, Medical Dictionary for Regulatory Activities.

^a^ Prophylactic G‐CSF was not given to dose‐escalation patients until after the first two cohorts who experienced exacerbated neutropenia.

Throughout the study, 61% of patients required some dose delay in study treatment due to either AEs, logistical barriers, or both. Approximately half (57%) of all patients experienced a delay in filanesib dosing due to AEs or abnormal laboratory findings, while 32% of all patients experienced a delay for reasons such as scheduling, holidays, or planned surgeries. Delays in bortezomib generally occurred alongside filanesib delays, with 57% of patients experiencing AE or laboratory‐related delays and 29% experiencing logistical delays. Half of expansion phase patients required dose reductions in filanesib, while 43% of patients required dose reductions in bortezomib. The most frequent AEs leading to dose reduction of filanesib were thrombocytopenia and pneumonia (each 7%), while peripheral neuropathy, neutropenia, and thrombocytopenia (each 7%) were the most frequent causes for dose reduction of bortezomib. Four patients (14%) discontinued study treatment due to AEs (amylase and lipase elevation related to bortezomib and filanesib, peripheral neuropathy related to bortezomib, neutropenia related to filanesib that resolved, and unintentional overdose of filanesib).

Only one death was attributable to AEs in a schedule 2 patient who died shortly after receiving the first dose of filanesib. The cause of death was suspected filanesib overdose with subsequent Steven‐Johnson Syndrome and septic shock, effects assessed as related to study treatment. The suspicion of the overdose was based on clinical symptoms and analysis of a pharmacokinetic blood sample suggesting an administered dose 5–10 times the intended dose. The site was inspected by Array CQA, IRB, and the FDA following the incident.

### 3.3 Efficacy

**FIGURE 1:**
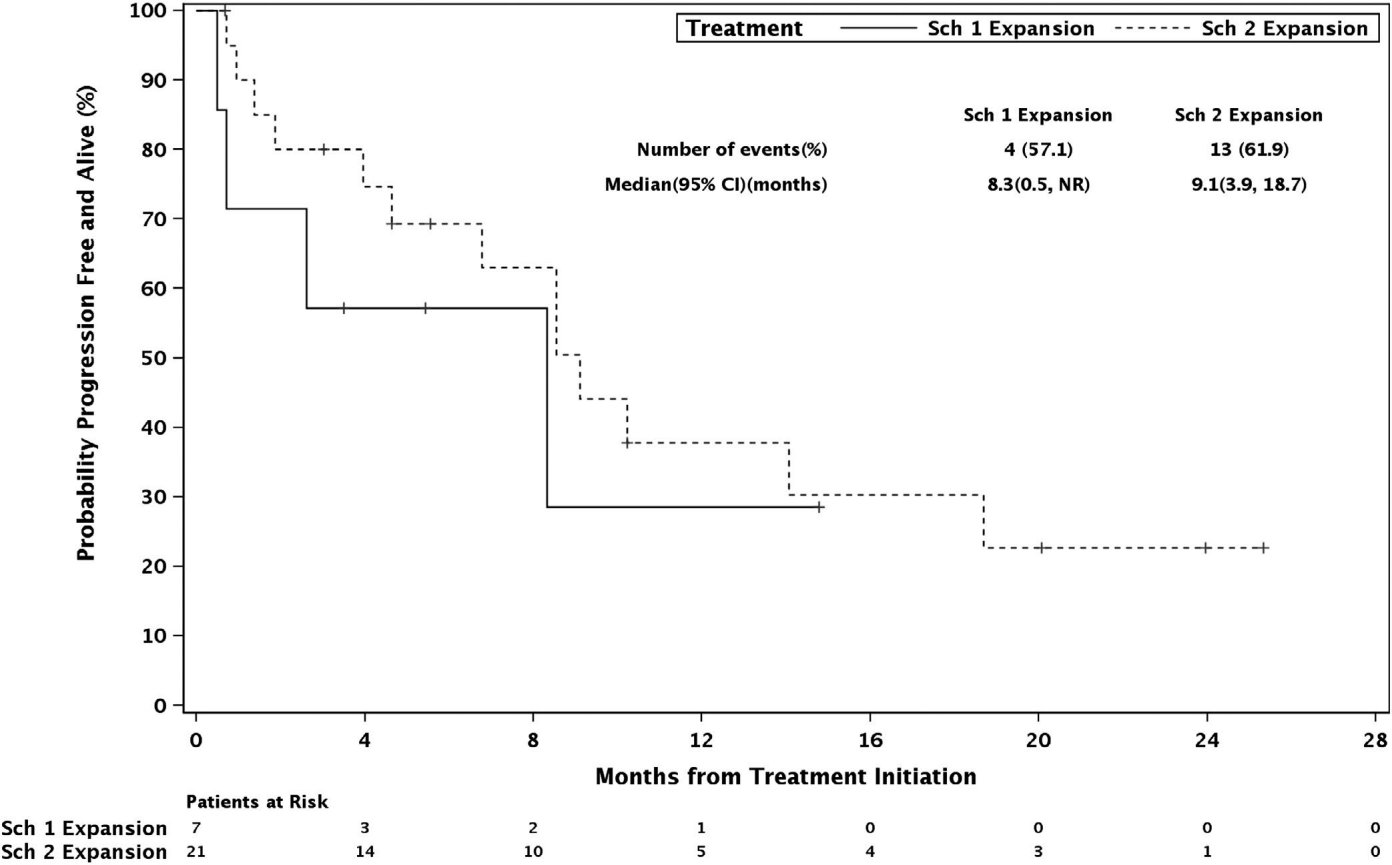
Progression‐free survival, dose‐expansion phase

There were 28 patients evaluable for efficacy in the expansion phase followed for a median of 6.3 months. The ORR of filanesib with weekly bortezomib and dexamethasone was 43% (Table 3). Responses were durable, and median DOR was not reached (range 2.8–≥23.7 months). Patients who attained a response generally did so in the first or second cycle, with a median time to response of 1.6 months (Table 4). The CBR was 63% and the DCR was 75%. Median PFS was 8.3 months for schedule 1 and 9.1 months for schedule 2 (Figure 1).

**TABLE 3:** Clinical response (response‐evaluable population)

	Dose‐escalation		Dose‐expansion		Phase 1 therapeutic total
Best disease response (%)	Schedule 1 therapeutic (*N* = 19)	Schedule 2 (*N* = 10)	Schedule 1 (*N* = 7)	Schedule 2 (*N* = 21)	(*N* = 57)
Stringent CR	1 (5)	0 (0)	0 (0)	0 (0)	1 (2)
CR	0 (0)	0 (0)	0 (0)	2 (10)	2 (4)
VGPR	4 (21)	1 (10)	0 (0)	2 (10)	7 (12)
PR	3 (16)	1 (10)	3 (43)	5 (24)	12 (21)
MR	3 (16)	2 (20)	1 (14)	4 (19)	10 (18)
SD ≥8 weeks	3 (16)	4 (40)	0 (0)	4 (19)	11 (19)
SD <8 weeks	3 (16)	1 (10)	1 (14)	1 (5)	6 (11)
PD	2 (11)	1 (10)	2 (29)	2 (10)	7 (12)
NE	0 (0)	0 (0)	0 (0)	1 (5)	1 (2)
ORR (≥PR)	8 (42)	2 (20)	3 (43)	9 (43)	22 (39)
CBR (≥MR)	11 (58)	4 (40)	4 (57)	13 (62)	32 (56)
DCR (≥SD>8 weeks)	14 (74)	8 (80)	4 (57)	17 (81)	43 (75)

Abbreviations: MR, minimal response; NE, not evaluable; PD, progressive disease; SD, stable disease.

**TABLE 4:** Time‐to‐event endpoints in the response‐evaluable population (dose‐expansion phase)

Parameter	Statistic	Schedule 1 expansion (*N* = 7)	Schedule 2 expansion (*N* = 21)	Expansion phase total (*N* = 28)
Time on study, month	*N*	7	21	28
	Median	4.5	9.2	6.3
	SD	4.89	7.51	7.07
	Min–Max	1.2–14.8	0.7–25.3	0.7–25.3
Time to first response (≥PR), month	*N*	3	9	12
Responders only	Median	3.4	1.6	1.6
	95% CI	0.7, 7.8	0.7, 1.9	0.7, 3.4
	Min–Max	0.7–7.8	0.7–4.4	0.7–4.4
Duration of response (≥PR), month	*N*	3	9	12
	Median	NR	NR	NR
	95% CI	2.8, NR	3.0, NR	6.6, NR
	Min–Max	2.8–6.9^a^	3.0–23.7^a^	2.8–23.7^a^

Abbreviations: 95% CI, 95% confidence interval; NR, not reached; PR, partial response.

^a^ Indicates censoring.

**FIGURE 2:**
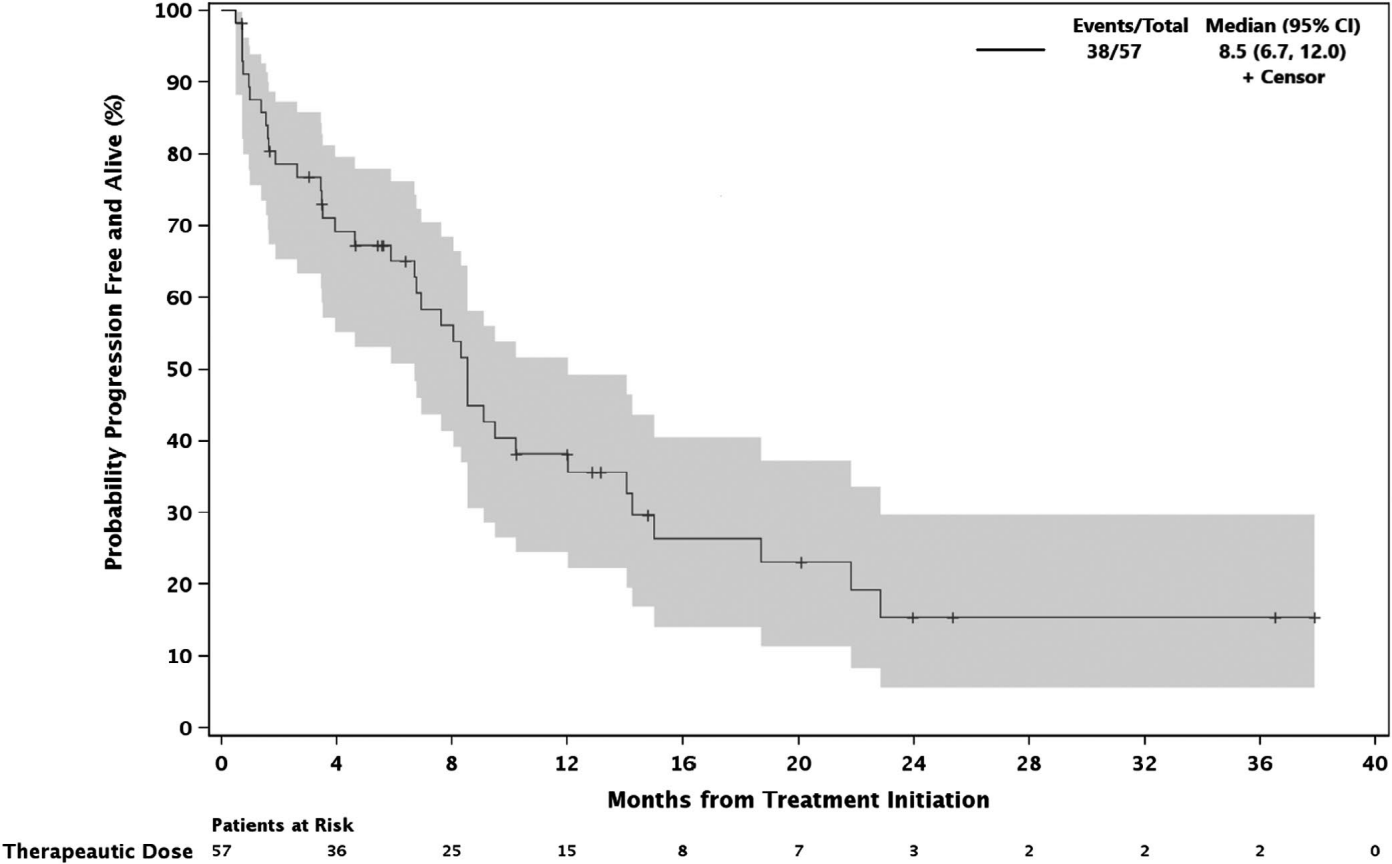
Progression‐free survival, phase 1 therapeutic dose patients

Therapeutic doses of filanesib are ≥1.25 mg/m^2^. Thus, we performed a post hoc analysis combining the above discussed 28 expansion phase patients with the previously published 29 dose‐escalation phase patients treated with therapeutic doses of the study drugs. These 57 patients, followed for a median of 8.5 months, had a median of three lines of prior therapy (range 1–11), and 32% (18/57) were PI‐refractory. The ORR was 39% with a median time to response among responders of 1.3 months and a median DOR of 18.0 months (range 2.8–≥34.9 months). The CBR was 56% and the DCR was 75% and median PFS was 8.5 months (Figure 2).

**FIGURE 3:**
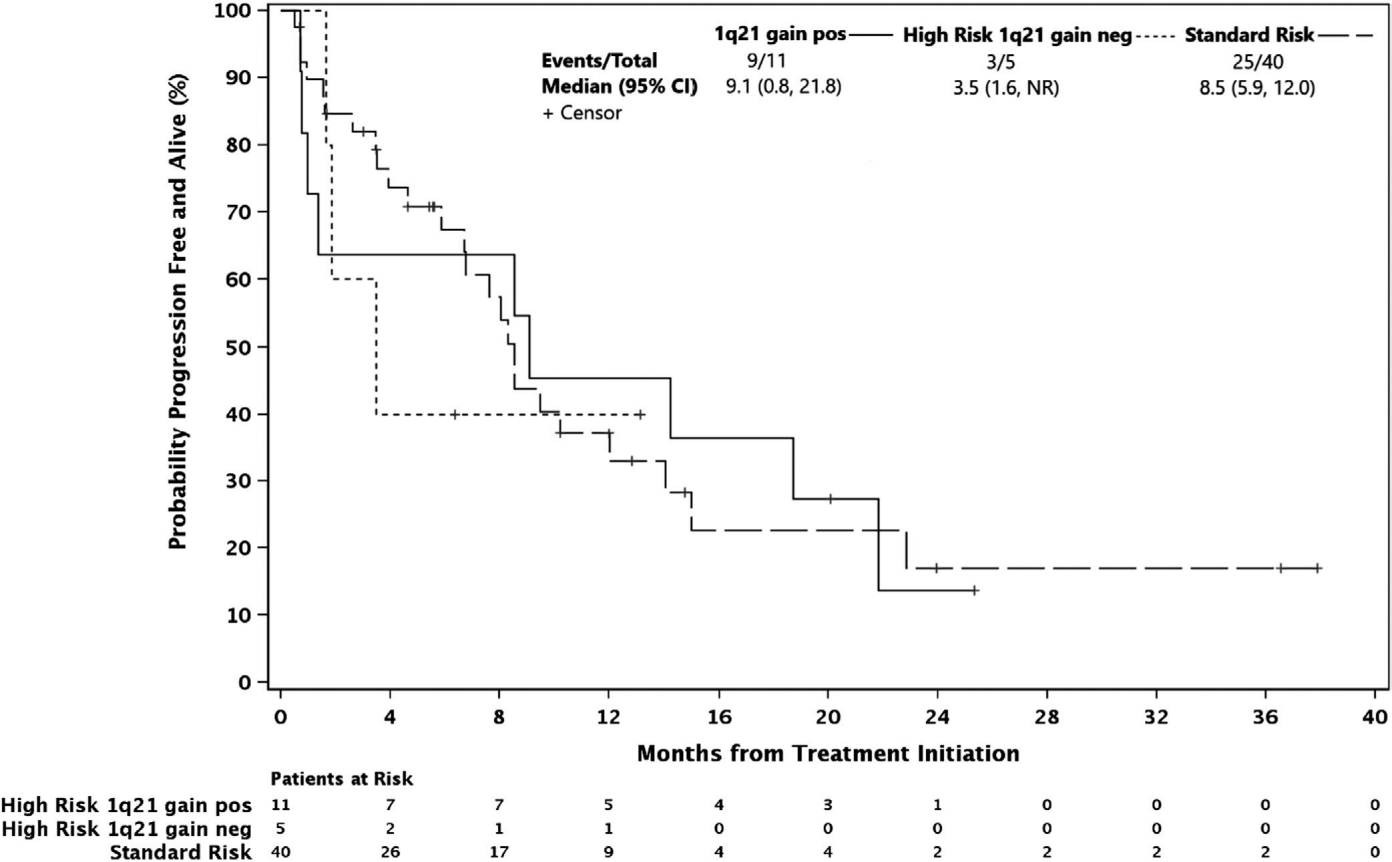
Progression‐free survival by 1q21 gain status, phase 1 therapeutic dose patients

**FIGURE 4:**
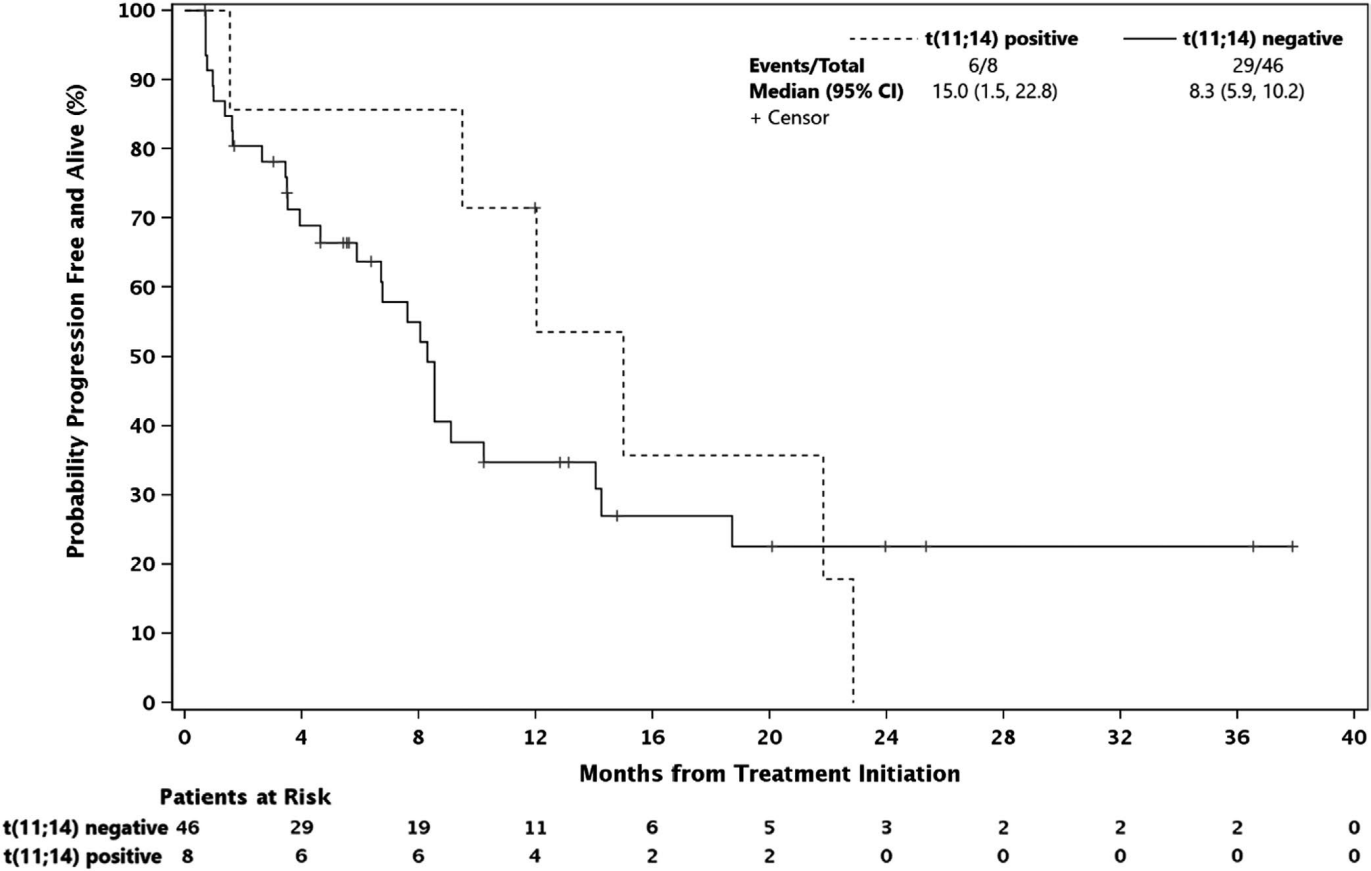
Progression‐free survival by t(11;14) status, phase 1 therapeutic dose patients

Among therapeutically dosed patients, efficacy results were comparable in patients with high‐risk cytogenetics and CrCl <60, with median PFS 8.5 and 8.5 months, respectively. Because patients with 1q21 gain demonstrated increased expression of MCL‐1 and sensitivity to MCL‐1 targeting, we stratified high‐risk patients based on whether they harbored this mutation.^12^, ^13^ The 11 1q21 gain patients had an ORR of 45% and median PFS 9.1 months, compared with 3.5 months in high risk patients without 1q21 gains (Figure 3). Among the 18 patients with PI‐refractory disease, ORR was 28% (3 VGPRs and 2 PRs), DCR was 67%, and median PFS was 5.9 months. Considering the role of BCL‐2‐like proteins in myeloma apoptosis, we specifically assessed the 8 t(11;14) patients and found an ORR of 33.3% and median PFS of 15.0 months (vs. 8.3 months in patients without the mutation, Figure 4).

**FIGURE 5:**
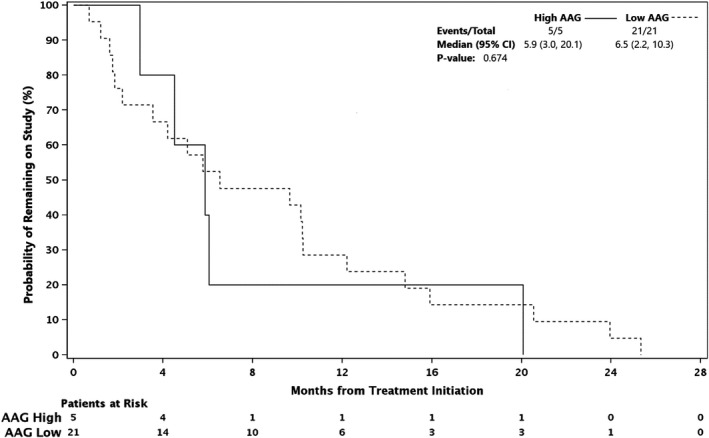
Time on study by AAG value

Stratification of patients by AAG (>110 mg/dl vs. ≤110 mg/dl) did not affect ORR or ToS (Table 5, Figure 5). Results from analysis of variance of filanesib and bortezomib concentrations indicated no clinically or statistically significant changes in mean filanesib concentration when co‐administered with bortezomib or vice‐versa (Tables S1 and S2).

**TABLE 5:** Time on study (dose‐expansion phase)

Group	No.	Time on study median (Min–Max), months
All patients	28	6.3 (0.7–25.3)
PI refractory	4	7.4 (1.6–20.8)
PI sensitive	19	6.6 (1.2–25.7)
Low AAG (≤110 mg/dl)	23	9.2 (0.7–25.7)
High AAG (>110 mg/dl)	5	5.9 (3.0–20.3)

Abbreviations: AAG, alpha 1‐acid glycoprotein; PI, proteasome inhibitor.

## 4 DISCUSSION

Filanesib is a highly selective, first‐in‐class inhibitor of KSP which has demonstrated synergistic antimyeloma activity in preclinical studies with both bortezomib and dexamethasone. Early data demonstrated the safety of filanesib in combination with bortezomib plus dexamethasone. After a dosing schedule was established for the combination, two expansion schedules were conducted with encouraging results.

Response rates of filanesib, bortezomib, and dexamethasone were comparable amongst patients receiving ≥1.25 mg/m^2^ of filanesib in both cohorts, with a combined ORR of 39%. This response rate is similar to that observed in the RETRIEVE study where bortezomib‐sensitive patients were retreated with bortezomib with or without dexamethasone and achieved an ORR of 40%. Importantly, however, RETRIEVE patients were treated with twice weekly 1.3 mg/m^2^ bortezomib‐dexamethasone in a 21‐day cycle.^18^ In contrast, the current study utilized only once weekly bortezomib 1.3 mg/m^2^ on days 1, 8, and 15 of a 28‐day cycle, i.e. 56% of the monthly dose of bortezomib given in RETRIEVE. Yet, responses in the current study were far more durable with a median DOR of 18.0 months compared with 6.5 months with bortezomib re‐treatment.^18^ Safety results were also comparable between the two studies, despite the addition of filanesib to the current regimen. The DOR also compares favorably with recently approved triplet regimens containing bortezomib and dexamethasone in patients with RRMM such as pomalidomide and daratumumab, with median DORs of 14 months and not reached at median follow‐up of 7.4 months, respectively.^19^, ^20^

The clinical efficacy of filanesib is further demonstrated in the PI‐refractory population where weekly bortezomib and dexamethasone alone would not be expected to produce results, whereas with the current regimen we observed 28% ORR and 5.9 months PFS. These results are similar to other agents approved in this refractory setting, which have produced ORRs and PFS ranging 23.7%–31% and 3.7–4.0 months, respectively.^21^, ^22^, ^23^, ^24^

Filanesib administration does not appear to affect the concentration of bortezomib or vice‐versa, and thus approved doses for the individual agents need not be adjusted for the combination. Plasma levels of AAG were also assessed. Though prior studies have found AAG a potentially useful biomarker of filanesib response, high or low AAG level did not seem to correlate with response to the current study's treatment, likely due to the fact that these patients could not be bortezomib refractory, potentially blunting the role of AAG.

Patients with t(11;14), in addition to high BCL‐2 expression, have levels of MCL‐1 mRNA expression comparable to other multiple myeloma subtypes.^12^ The outcomes of our regimen in the t(11;14) population are noteworthy given the PFS of 14.2 months. This PFS falls between the medians of 6.6 months with venetoclax monotherapy and not yet reached at median 18.7 months follow‐up with venetoclax, bortezomib, and dexamethasone in t(11;14) patients.^14^, ^25^ Patients in these groups had five median lines of therapy and one to three prior lines, respectively. Still, in our combination study, it is difficult to distinguish the roles of the proteasome inhibitors, which are also known to upregulate NOXA and Mcl‐1cleavage, from filanesib.^26^ Further investigation of filanesib activity in this group is warranted.

Notably, this regimen had comparable efficacy in patients with high‐risk cytogenetics with median PFS of 8.5 months. These results were driven by the 9.1 month PFS among patients with 1q21 gains, an aberration that typically confers inferior outcomes in bortezomib‐based regimens, while high‐risk patients without this mutation fared worse (3.4 months).^27^ Considering the higher levels of MCL‐1 expressed in 1q21 gain patients and their susceptibility to MCL‐1 targeting, further investigation of filanesib in this subset is warranted.^12^, ^13^

The combination of filanesib, bortezomib, and dexamethasone continues to show safety and encouraging activity in this dose‐expansion phase. Among all phase 1 patients who had received therapeutic doses of study drugs, a median DOR of 18.0 months including some patients remaining on treatment more than 35 months was observed among the 39% of responders. Investigation into the predictive value of t(11;14), 1q21 gain, AAG, and other biomarkers is needed before embarking on a randomized phase 3 study.

## CONFLICTS OF INTEREST

Jonathan L. Kaufman has received personal fees and acted as a paid consultant for Amgen, Bristol Meyers Squib, Celgene, Janssen, Sanofi/Genzyme, Takeda, and Tecnopharma and acts as a member of the Advisory Board for Incyte, Karyopharm, Pharmacyclics, and TG Therapeutics. Jeffrey A. Zonder receives research support and personal fees from Celgene and Bristol Meyers Squib and acts as a member of the Advisory Board for Takeda, Amgen, Oncopeptides, Janssen, Intellia, Alnylam, and Caelum. Selena Rush is a former employee of Pfizer and was an employee of Array at the time of the study. Brian J. Tunquist is a current employee of Pfizer and has one approved patent (US Patent 9,561,214) and one patent currently pending (US Patent Application 2018/0338958 [Method of Treatment Using Inhibitors of Mitosis]). Ajai Chari reports grants and personal fees from Janssen, Celgene, Novartis Pharmaceuticals, Seattle Genetics, Millenium/Takeda, and Amgen, personal fees from Bristol Myers Squibb, Karyopharm, Sanofi, Oncopeptides, and Antengene and grants from Pharmacyclics for work performed outside of the current study.

The current study was conducted in accordance with International Conference on Harmonisation of Technical Requirements for Registration of Pharmaceuticals for Human Use Good Clinical Practice guidelines and regulations. The study was approved by the Institutional Review Boards of all participating centers, and patients provided written informed consent. This study was registered at www. ClinicalTrials.gov with identifier NCT01248923.

## AUTHOR CONTRIBUTIONS

Ajai Chari, Brian J. Tunquist, Selena A. Rush, and Jonathan L Kaufman conceived and designed the study. Jonathan L. Kaufman, Myo Htut, Manish Agrawal, Amitabaha Mazumder, Robert F. Cornell, Jeffrey A. Zonder, Joseph W. Fay, Manuel R. Modiano, Selena A. Rush, Brian J. Tunquist, and Ajai Chari collected data. Darren Pan, Jonathan L. Kaufman, Myo Htut, Manish Agrawal, Amitabaha Mazumder, Robert F. Cornell, Jeffrey A. Zonder, Joseph W. Fay, Manuel R. Modiano, Erin L. Moshier, Selena A. Rush, Brian J. Tunquist, and Ajai Chari interpreted and analyzed data and performed research. Darren Pan, Erin L. Moshier, Selena A. Rush, and Ajai Chari performed statistical analysis. Darren Pan and Ajai Chari wrote the article. All authors read the final article, contributed to essential edits and revisions, and approved the final version for submission.

## ETHICAL APPROVAL

The current study was conducted in accordance with International Conference on Harmonisation of Technical Requirements for Registration of Pharmaceuticals for Human Use Good Clinical Practice guidelines and regulations. The study was approved by the Institutional Review Boards of all participating centers, and patients provided written informed consent. This study was registered at www. ClinicalTrials.gov with identifier NCT01248923.

## Supporting information

Table S1‐S2

## Data Availability

The data that support the findings of this study are available from the corresponding author upon reasonable request.
