# Supplementary material for: Filanesib plus bortezomib and dexamethasone in relapsed/refractory t(11;14) and 1q21 gain multiple myeloma
Source: Cancer Med. 2021 Dec 17;11(2):358–70. doi: 10.1002/cam4.4451 (PMC8729045; doi:10.1002/cam4.4451)
Supplement: Supplementary file 1 — Table S1‐S2 [file CAM4-11-358-s001.docx]

**SUPPLEMENTARY TABLE 1.** Effect of Filanesib on Bortezomib Pharmacokinetics

| Ratio | N | Geometric Mean Ratio | Geometric CV (%) | Median | Min, Max |
| --- | --- | --- | --- | --- | --- |
| Schedule 1 (Expansion) | | | | | |
| Day 1 to 8 | 5 | 0.795 | 24.5 | 0.723 | 0.597, 1.09 |
| Day 15 to 8 | 7 | 0.982 | 34.7 | 1.02 | 0.614, 1.50 |
| Schedule 2 (Expansion) | | | | | |
| Day 1 to 8 | 19 | 0.712 | 50.6 | 0.724 | 0.213, 1.69 |
| Day 15 to 8 | 19 | 1.01 | 62.5 | 1.05 | 0.201, 2.42 |

**SUPPLEMENTARY TABLE 2.** Effect of Bortezomib on Filanesib Pharmacokinetics

| Ratio | N | Geometric Mean Ratio | Geometric CV (%) | Median | Min, Max |
| --- | --- | --- | --- | --- | --- |
| Day 2 to 1 | 7* | 1.12 | 76.4 | 1.41 | 0.258, 2.01 |
| Day 16 to 15 | 7 | 1.28 | 16.4 | 1.23 | 1.02, 1.65 |

Abbreviations: CV = coefficient of variation

*Limited to n = 7, as only schedule 1 of the expansion phase included days when filanesib was dosed alone (Day 2 and Day 16). Due to small sample size, an analysis of variance of the exposure ratios was not performed. However, no clinically significant changes in mean filanesib concentrations were seen when co-administered with bortezomib.
